# How Does Job Dissatisfaction Fuel Nurse Exit, Voice, Loyalty, and Neglect (EVLN) Behaviours? Insights From Dynamic Game Theory and a Cross‐Sectional Survey

**DOI:** 10.1002/nop2.70313

**Published:** 2025-10-08

**Authors:** Jackie Zhanbiao Li, Ming Chen, Yingqian Lao, Wanqing Hu

**Affiliations:** ^1^ Affiliated Banan Hospital of Chongqing Medical University Chongqing China; ^2^ Hong Kong Metropolitan University School of Nursing and Health Sciences Hong Kong China; ^3^ Department of Rehabilitation Medicine The Affiliated BenQ Hospital of Nanjing Medical University Nanjing China; ^4^ The First Affiliated Hospital of Guilin Medical University Guilin China

**Keywords:** China, decision theory, health workforce, job dissatisfaction, nurses, organisational behaviour

## Abstract

**Aims:**

This study explores how nurses' job dissatisfaction influences their behavioural responses—exit, voice, loyalty, and neglect (EVLN)—within the framework of organisational behaviour, focusing on the Chinese cultural context using dynamic game theory. The goal is to provide insights for reducing nurse shortages.

**Design:**

A cross‐sectional quantitative design was employed to assess the relationships between nurses' job dissatisfaction and EVLN behaviours in public hospitals in Chongqing, China, between January and May 2024.

**Methods:**

Data were collected using a structured questionnaire administered to registered nurses across tertiary public hospitals. The study combined Farrell's EVLN framework with dynamic game theory to analyse behavioural evolution. Quantitative analysis included descriptive statistics, reliability checks, correlation tests, multiple regression analyses, robustness checks, and heterogeneity analysis.

**Results:**

This study involved 1132 registered nurses, primarily female (94.88%), aged 20–39. Findings show that job dissatisfaction is positively correlated with exit (*β* = 0.3924, *p* < 0.01) and neglect (*β* = 0.6603, *p* < 0.01) behaviours, and negatively correlated with voice (*β* = −0.1483, *p* < 0.01) and loyalty (*β* = −0.1714, *p* < 0.01). Gender and education level significantly influenced the relationship between dissatisfaction and EVLN behaviours, while age and marital status showed partial heterogeneity. Dynamic game modelling revealed that nurses initially showing loyalty may shift to voice if dissatisfaction persists, and if voice is ignored, they may either transition to exit or move gradually to neglect.

**Conclusion:**

This study contributes to nursing management by applying dynamic game theory to the EVLN framework, revealing the evolutionary mechanisms through which nurses respond to job dissatisfaction and highlighting the cultural specificity of these behaviours in China's healthcare setting.

**Implications for the Profession:**

Understanding the dynamic responses to job dissatisfaction can assist hospital administrators in developing strategies that promote nurse retention, encourage voice and loyalty behaviours, and mitigate exit and neglect.

**Impact:**

This study enriches the application of EVLN theory in China and Greater China, supporting hospital management and optimizing nursing strategies for sustainable hospital development.

**Patient or Public Contribution:**

No direct patient or public contribution.

## 1 Introduction

Nursing plays a vital role in global medical care and has significantly contributed to the provision of high‐quality medical services (Tamata and Mohammadnezhad 2023). A sufficient number of nurses is essential for strengthening the medical care sector, expanding health care coverage, and achieving the WHO's sustainable development health goals (Alameddine et al. 2017). Despite the crucial role of nursing in medical care, one of the major challenges facing the global medical system today is the severe shortage of nurses, which adversely affects the quality of care and the health and well‐being of populations worldwide (Wu, Xue, and Zhang 2024; Yahyaei et al. 2022). This shortage impacts over 1 billion people, particularly vulnerable groups such as women and children, who are in urgent need of high‐quality medical services (Aluko et al. 2019; Chang et al. 2024). A primary reason for this shortage is the high turnover rate among nurses due to job dissatisfaction. If left unaddressed, this issue will perpetuate a vicious cycle of continued nursing shortages, declining care quality, and an inability to meet the health needs of global populations effectively (Matsuo et al. 2021; Wong 2024). Therefore, addressing the root causes of the nurse shortage and understanding the underlying reasons for nurse attrition have become critical topics in both academia and practice. Investigating and analysing nurses' behavioural responses to job dissatisfaction is a key step in addressing this issue.

As a model for measuring behavioural responses to job dissatisfaction, EVLN (exit, voice, loyalty, and neglect) has become a key framework for scholars globally to study stakeholder behaviour in situations of dissatisfaction. It has been widely applied in various research areas, such as psychological contracts (Cheng et al. 2025; Hu et al. 2023), human resource management (Barry and Wilkinson 2022; Nair 2025), and conflict management (Lee and Varon 2020; Park and Gong 2025). However, the use of the EVLN model to examine nurses' behavioural responses to job dissatisfaction within the medical system remains underexplored. This study employs a mixed‐methods approach to analyse the relationship between nurses' job dissatisfaction and their corresponding EVLN behaviours, as well as the evolution of these behaviours in response to job dissatisfaction.

To establish the link between nurses' job dissatisfaction and the EVLN behaviours, we utilised a questionnaire‐based approach to collect data on nurses' behavioural responses in tertiary public hospitals in Chongqing's urban and county districts from January to May 2024. Dynamic game theory was then applied to investigate the relationship between nurses' job dissatisfaction and their EVLN behaviours, and to explore the evolution of these behaviours over time.

This study makes significant contributions to the existing literature on the EVLN model triggered by job dissatisfaction in several key aspects. First of all, by incorporating the Chinese cultural context, it introduces dynamic game theory into the EVLN behaviour analysis framework for the first time, exploring the relationship between nurses' job dissatisfaction and EVLN behaviours. Through this analytical framework, the study not only enriches the application of the EVLN theory in China and the Greater China region but also provides empirical support for hospital management, particularly in addressing nurses' job dissatisfaction. Unlike most existing research, which focuses on Western countries, this study examines the impact of the more pronounced hierarchical structure in Chinese culture on nurses' behavioural choices when faced with job dissatisfaction, especially regarding the expression of “voice” behaviour. In China's collectivist culture, nurses tend to cope with work pressure through “loyalty” rather than directly expressing dissatisfaction, reflecting the significant cultural influence on EVLN behaviours.

Secondly, this study expands the impact of job dissatisfaction on employee behaviour, particularly in the context of hospital nurses, enhancing the theoretical framework between nurses' job dissatisfaction and EVLN behaviours, and further strengthening the research on the influence of EVLN behaviours on hospital management. In addition, this research bridges the fields of psychology, behavioural economics, and hospital management, offering new perspectives and insights to expand the literature in these areas. Finally, by innovatively applying dynamic game theory to examine the evolutionary mechanisms of EVLN behaviours triggered by nurses' job dissatisfaction, this study provides new theoretical support for the behavioural evolution models in hospital management.

Our study is organised into six sections: the second section presents the theoretical foundation and research hypotheses, the third covers the research design, the fourth reports the results, the fifth explores heterogeneity and the evolution of EVLN behaviours, and the sixth provides the conclusion.

## 2 The Theoretical Basis and Research Hypotheses

### 2.1 The Theoretical Basis of EVLN Model

**FIGURE 1:**
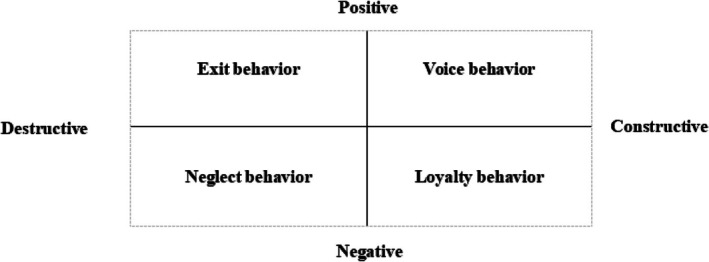
The Exit, Voice, Loyalty, and Neglect (EVLN) individual response categorization model. *Source:* Farrell (1983)

The development of the EVLN model has progressed through three crucial stages. First, the EVL theory, proposed by Hirschman (1970), suggests that when individuals are dissatisfied with the functioning of a social system, they typically exhibit three behaviours—exit, voice, or loyalty—collectively known as the EVL theory. Hirschman also elaborated on the specific concepts underlying these three behaviours. Second, Kolarska and Aldrich (1980) and Rusbult and Zembrodt (1982) further advanced this theory by introducing the EVLN model, explicitly identifying the behaviour of “neglect” for the first time. In their research, neglect was characterised by a lack of concern, carelessness, and irresponsible behaviour. Finally, Farrell (1983) refined and expanded the EVLN theory by formally incorporating neglect behaviour and organising the four behaviours—exit, voice, loyalty, and neglect—along two dimensions: constructive‐destructive and active‐passive. This development resulted in a more comprehensive EVLN model, as illustrated in Figure 1 below.

The EVLN model distinguishes between constructive behaviours (loyalty, voice) and destructive ones (exit, neglect). The second dimension is the active–passive (or positive–negative) dimension. Exit and voice behaviours are viewed as active forms of expression, indicating a willingness to address job dissatisfaction. Conversely, neglect and loyalty behaviours are considered passive and negative forms of expression. According to Farrell, exit refers to the voluntary act of leaving a job, whether by exiting the company or seeking new employment to escape an unsatisfactory position. Voice emphasises the importance of employee participation in addressing organisational decline and restoring previous performance levels, functioning as a key recovery mechanism that requires collective employee involvement. Loyalty reflects an individual's patience in awaiting positive changes within the organisation, with the belief that the organisation's decisions are ultimately correct, consistent with the findings of Williams et al. (2001). Neglect, on the other hand, refers to behaviours such as disengagement, absenteeism, and increased error rates that arise from job dissatisfaction, leading to reduced productivity and serving as a warning signal to management.

### 2.2 Research Hypothesis on the Relationship Between Job Dissatisfaction and EVLN Behaviours

#### 2.2.1 Job Dissatisfaction and Exit Behaviour

Exit behaviours among employees primarily refer to inter‐organisational mobility, intra‐organisational mobility, and cognitive activities preceding such mobility (Farrell 1983). Research has demonstrated that when employees experience job dissatisfaction due to both internal and external factors, they often choose to exit (Hörberg et al. 2023; Lee et al. 2025). According to dynamic game theory, when conflicts of interest arise between employees and organisations and the organisation fails to implement effective measures to address employee demands and allocate necessary resources, employees will feel dissatisfaction and disappointment during the interaction. To maximise their benefits, employees may seek to change their environment (Ke et al. 2022; Lu, Yang, et al. 2025). In such cases, exit behaviour serves as an effective means of resolution (Grandey and Cropanzano 1999). Numerous empirical studies have shown that when job satisfaction is low, exiting to protect one's resources is a rational choice (Chen et al. 2023; Hidaka et al. 2025). However, in contrast to Western cultural perspectives, in the Chinese cultural context, the relationship between nurses' job dissatisfaction and “exit” behaviour may be profoundly influenced by cultural and social structures (He et al. 2024; Kong et al. 2024). Chinese culture emphasises collectivism, family values, and the maintenance of social relationships, and nurses often possess a strong sense of “loyalty.” This cultural inclination may lead hospital nurses to initially attempt to cope with job dissatisfaction through “loyalty” or “silence,” rather than immediately resorting to exit behaviour (Zhang, Ma, et al. 2025). However, as conflicts of interest between nurses and the organisation escalate, and if the organisation fails to effectively respond to their needs over time, nurses may become disappointed with the organisation during the game‐theoretic process. Eventually, when their disappointment reaches a certain threshold, they may decide to exit the organisation (Bruyneel et al. 2025; Li and Li 2024). Therefore, based on the aforementioned theory, when hospital nurses experience job dissatisfaction, they are likely to exhibit exit behaviour. From this, we propose Hypothesis 1a:

Hypothesis 1a *Nurses' job dissatisfaction is positively related to exit behaviour*.

#### 2.2.2 Job Dissatisfaction and Voice Behaviour

Voice behaviours are regarded as a form of constructive communication that allows individuals to express opinions thoughtfully and improve organisational conditions (Kim and Rim 2023). Voice is an autonomous behaviour that reflects the exercise of individual rights and is typically a result of careful consideration, weighing the potential benefits and drawbacks (McKeever et al. 2023). Employee‐centered voice behaviours involve actively expressing opinions when employees perceive a task as low‐risk and efficient and when they believe that voicing their concerns will result in obtaining deserved resources. However, when organisational responses do not support these behaviours, employees may choose to remain silent (Shipton et al. 2024). In the Chinese cultural context, collectivism and respect for authority emphasise the maintenance of harmonious organisational relationships, which may lead nurses to prefer silence when faced with job dissatisfaction (Zhang, Ma, et al. 2025). In Chinese society, nurses often prioritise stable career development and their relationships with superiors (Cao et al. 2025). Therefore, despite the potential benefits of voice behaviour in improving the organisational environment, nurses may choose silence for fear that expressing dissatisfaction could negatively impact their career progression and interpersonal relationships. According to dynamic game theory, conflicts between employees and organisations arising from work‐related issues lead employees to evaluate the risks and rewards of voice behaviour, while organisations respond based on employee demands. When employees perceive that expressing their concerns may lead to potential losses—such as diminished access to valuable personal resources or career opportunities—and when the expected benefits do not outweigh these risks, they often opt for silence (Haurie et al. 2012; Başar and Zaccour 2018). In Chinese culture, the emphasis on “face” and “authority” influences the dynamic between nurses and organisations. During the game‐theoretic process, expressing dissatisfaction is often perceived as disruptive and disrespectful to superiors. This cultural norm leads nurses to prioritise avoiding conflict and opting for silence over engaging in voice behaviour when faced with job dissatisfaction (Lu, Yang, et al. 2025; Yang et al. 2025). Previous empirical studies have shown that voice behaviour can have negative consequences for the speaker, as they may be seen as troublemakers, which can affect their career progression and access to personal resources (Detert and Burris 2007; Lu, Jin, et al. 2025). In the Chinese work environment, silence is often considered the safest and most effective strategy for survival (Lehmann and Wagoner 2025). Therefore, when nurses in hospital experience job dissatisfaction, they often avoid voice behaviour to protect their careers and opportunities for advancement. Based on this, we propose Hypothesis 1b:

Hypothesis 1b *Nurses' job dissatisfaction is negatively related to voice behaviour*.

#### 2.2.3 Job Dissatisfaction and Loyalty Behaviour

Loyalty behaviours refer to the actions of employees who adhere to strong professional ethics and either passively or optimistically expect the organisation to improve itself and make necessary adjustments (Rusbult et al. 1988; Allen and Grisaffe 2001). Research shows that subordinates are more likely to exhibit loyalty toward supervisors or organisations that prioritise their vital interests and well‐being. Employees believe that such supervisors or organisations will act in their best interest, fostering a sense of belonging (Jung and Choi 2025; Nguyen and Ha 2023). In the Chinese cultural context, particularly in hospital environments characterised by strong collectivist values, loyalty behaviour is often viewed as a virtue. Nurses typically expect to gain organisational recognition through the demonstration of loyalty. This tendency becomes more pronounced when viewed through the lens of dynamic game theory (Maksim and Śliwicki 2025; Setyanto and Arafah 2025). Dynamic game theory explains how unmet expectations can reduce motivation and loyalty over time. Conversely, if employees' resource investments go unrewarded, they become unwilling to invest further, leading to a decline in organisational loyalty. In the collectivist culture of China, dynamic game theory exacerbates the relationship between nurses' job dissatisfaction and loyalty (Zhong et al. 2025). Because nurses highly value social relationships and organisational status, when an organisation fails to fulfil its promises or provide adequate support, nurses perceive a lack of return on their investment of resources. This leads to profound disappointment, making them reluctant to invest in any further organisational commitments. This imbalance ultimately results in nurses withdrawing their loyalty from the organisation. Therefore, when nurses in hospitals experience job dissatisfaction, they are likely to develop negative emotions and reduce their organisational loyalty to the hospital. Based on this, we propose Hypothesis 1c:

Hypothesis 1c *Nurses' job dissatisfaction is negatively related to loyalty behaviour*.

#### 2.2.4 Job Dissatisfaction and Neglect Behaviour

Employee neglect behaviours refer to actions where employees passively worsen the situation by being consistently late, absent, or using work time for personal matters, ultimately leading to a state of disengagement or slacking (Rusbult et al. 1988; Grifno et al. 2025). Neglect behaviour is often viewed as a manifestation of stress relief. When employees are dissatisfied with their work, they respond negatively to stressors (Rai and Koodamara 2025). From the perspective of dynamic game theory, conflicts between employees and organisations, particularly when employees feel that their resource investments are not reciprocated, can result in dissatisfaction. In these situations, the organisation should promptly address employee demands during the dynamic game process. Failure to do so can lead employees to feel resource‐threatened, resulting in unmet psychological and physical needs and triggering passive reactions, such as neglect behaviours (Haurie et al. 2012; Başar and Zaccour 2018; Ke et al. 2022). In the context of Chinese culture, nurses, who strongly embody collectivist values and loyalty, tend to avoid directly expressing dissatisfaction when faced with organisational issues. During the dynamic game process, nurses often choose to remain silent and avoid conflict with their superiors in order to protect their social relationships and maintain loyalty to the organisation and authority (Jungst and Verbeeck 2025; Lu, Yang, et al. 2025). However, when nurses' long‐term resource investment is not adequately reciprocated by the organisation, they may experience feelings of disappointment and frustration, which ultimately manifest in neglect behaviours aimed at avoiding further conflict or emotional exhaustion (Zhang, Huang, et al. 2025). Neglect behaviours, such as prolonged absenteeism, taking breaks, or adopting a passive attitude toward work tasks, not only reflect employees' responses to job dissatisfaction but also serve as a mechanism for self‐protection and stress relief (Khakpour 2025; Rai and Koodamara 2025). Therefore, when nurses in hospitals are dissatisfied with their work, they may adopt neglect behaviours as a passive means of self‐preservation. Based on this, we propose Hypothesis 1d:

Hypothesis 1d *Nurses' job dissatisfaction is positively related to neglect behaviour*.

#### 2.2.5 The Evolution of EVLN Behaviours Affected by Nurses' Job Dissatisfaction

Based on the aforementioned assumptions and analysis, it can be concluded that employee job dissatisfaction leads to the emergence of EVLN behaviours. Specifically, employee job dissatisfaction (including internal and external) is positively correlated with exit and neglect behaviours, while negatively correlated with voice and loyalty behaviours. However, the question remains: how do these EVLN behaviours evolve?

According to dynamic game theory (Haurie et al. 2012; Başar and Zaccour 2018; Ke et al. 2022), when there is an imbalance between resource allocation and compensation between employees and organisations, gaming behaviour arises. In the Chinese cultural context, nurses, as part of the organisation's collectivist culture, tend to endure and remain silent when faced with job dissatisfaction in order to avoid direct conflict with the organisation or superiors. This reflects the concepts of “loyalty” and “face” in Eastern culture (Zhong et al. 2025). However, when job dissatisfaction accumulates to a certain level, particularly due to unequal resource distribution and inadequate compensation, nurses' loyalty behaviour may be challenged, ultimately triggering a dynamic game process in which EVLN behaviours evolve in response to nurses' job dissatisfaction (Smith‐Miller and Cline 2025). Specifically, when employees are dissatisfied with the allocation of resources at work, a rational organisation, aiming to minimise losses, will strive to meet employees' needs as much as possible during the game‐theoretic process. This, in turn, increases employees' loyalty and alleviates job dissatisfaction (Rusbult et al. 1988). However, as employees' tenure increases, the initial resource allocation by the organisation may no longer meet their needs. Consequently, employees may demand higher compensation. If the organisation fails to adjust its measures promptly during the game‐theoretic process to meet these demands, employees will initially remain silent, hoping for an improvement before taking further action (Li, Qiu, et al. 2025). In the Chinese cultural context, nurses typically tend to avoid conflict with superiors to maintain “face” and harmonious workplace relationships. Therefore, they often delay directly expressing dissatisfaction and attempt to resolve the issue through silent waiting (Wu, Mao, et al. 2024). However, if the situation does not improve over time, they may choose voice behaviour rather than immediately opting for exit (Hirschman 1970; John 2017).

Following employee voice, when the organisation responds to job dissatisfaction with only minimal attention, employees may experience mild disappointment during the game‐theoretic process. This disappointment often manifests as disengagement in their work performance and a growing motivation to leave the organisation (Khan et al. 2025). In the Chinese work environment, particularly in hospitals, which are deeply influenced by collectivist cultural values, such disappointment may be culturally suppressed. Although nurses may feel dissatisfied with their work, they are more likely to endure and reserve their dissatisfaction until the issue becomes irreconcilable, at which point they may choose to leave (Zhou et al. 2025). Thus, when organisational dissatisfaction remains unaddressed, employees may experience profound disappointment, ultimately leading to their departure from the organisation (Farrell 1983; Treuren 2024). This suggests that if voice behaviour is not acknowledged by the organisation, it may initially lead to neglect behaviour, which may later evolve into exit behaviour. Alternatively, voice behaviour may directly result in exit behaviour (Lin et al. 2010).

In summary, the evolution of EVLN behaviours in the dynamic game between employees and organisations unfolds as follows: Employees initially adopt loyalty behaviour in response to job dissatisfaction (both internal and external). If the dissatisfaction persists, they shift to voice behaviour. If voice behaviour is not addressed, employees may either directly choose exit behaviour or transition from neglect to exit behaviour. Thus, the evolution of EVLN behaviours among nurses in hospitals, driven by job dissatisfaction, follows this progression. Based on this framework, we propose Hypotheses 2a, 2b, 2c and 2d.

Hypothesis 2a *Nurses' loyalty behaviour has a positive impact on voice behaviour*.

Hypothesis 2b *Nurses' voice behaviour has a positive impact on neglect behaviour*.

Hypothesis 2c *Nurses' neglect behaviour has a positive impact on exit behaviour*.

Hypothesis 2d *Nurses' voice behaviour has a positive impact on exit behaviour*.

**FIGURE 2:**
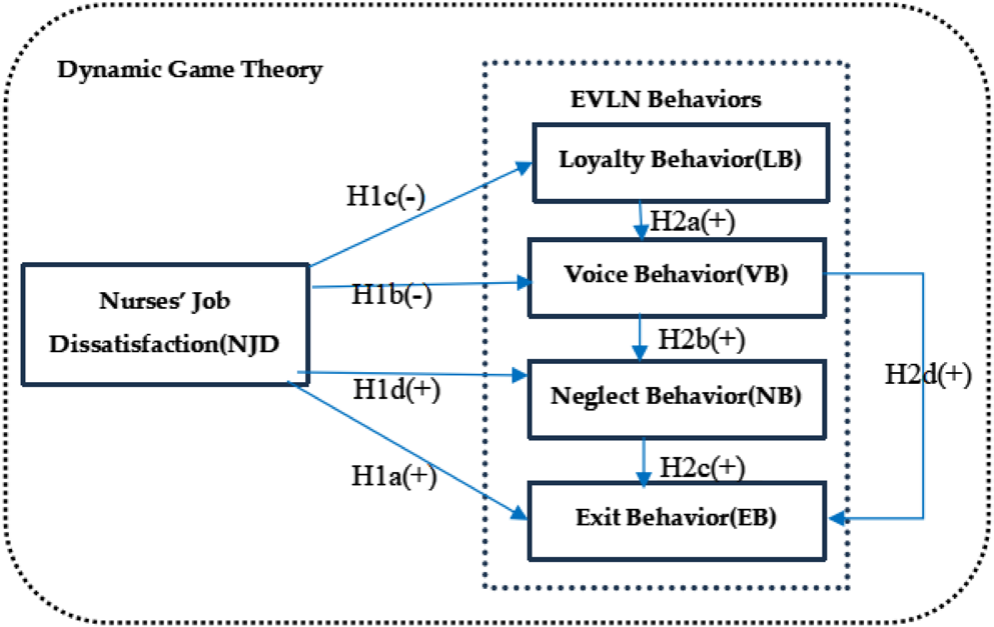
Conceptual framework on nurses' job dissatisfaction and Exit, Voice, Loyalty, and Neglect (EVLN) behaviours. Hypothesis 1a: Nurses' job dissatisfaction is positively related to exit behaviour; Hypothesis 1b: Nurses' job dissatisfaction is negatively related to voice behaviour; Hypothesis 1d: Nurses' job dissatisfaction is positively related to neglect behaviour; Hypothesis 2a: Nurses' loyalty behaviour has a positive impact on voice behaviour; Hypothesis 2b: Nurses' voice behaviour has a positive impact on neglect behaviour; Hypothesis 2c: Nurses' neglect behaviour has a positive impact on exit behaviour; Hypothesis 2d: Nurses' voice behaviour has a positive impact on exit behaviour. (−): Negative; (+): Positive.

Based on the aforementioned analysis, we determined the impact of nurses' job dissatisfaction on the relationships among various EVLN behaviours and developed a theoretical framework depicting the evolution of these behaviours (see Figure 2).

## 3 Research Design

### 3.1 Sample Selection

As the tertiary public hospitals in Chongqing play a significant role within China's healthcare system, encompassing various types of nursing personnel, they provide a comprehensive reflection of nurses' job dissatisfaction and their EVLN (Exit, Voice, Loyalty, and Neglect) behaviour responses in Chinese hospitals. Tertiary hospitals generally undertake more complex medical tasks and are better equipped with resources. Furthermore, as Chongqing represents a key region in central and western China, the findings from this study not only offer valuable data to explore regional differences in healthcare resources across China, but also have high external validity within the broader Chinese healthcare context. Therefore, this study uses nursing staff from tertiary public hospitals in the central districts and surrounding counties of Chongqing (qualified nurses holding a professional nursing license) as the research sample to examine their EVLN responses in situations of job dissatisfaction. Data collection was conducted from January to May 2024 through anonymous surveys, ensuring the comprehensiveness, objectivity, fairness, and rigor of the study. With the help of friends, classmates, and professionals in the medical field, a total of 1381 questionnaires were distributed, with 1219 returned, resulting in a response rate of 88.27%. After screening, questionnaires completed by trainees, interns, or those with incomplete or inaccurate responses were excluded, leaving 1132 valid questionnaires, yielding an effective response rate of 81.97%. The basic information of the study sample includes variables such as gender, age, marital status, and education level, with women comprising 94.88% of the sample. The majority of the participants were aged between 20 and 39, and most had a bachelor's degree or higher. These statistical data provide a solid foundation for the subsequent analysis of nurses' job dissatisfaction and their EVLN behavioural responses (see Table 1).

**TABLE 1:** Sample descriptive statistics.

Sociometric variables	Project	*N*	Percentage (%)
Gender (*N* = 1132)	Male	58	5.12
	Female	1074	94.88
Age (years) (*N* = 1132)	< 20	5	0.44
	20–29	431	38.07
	30–39	540	47.70
	≥ 40	156	13.78
Marital status (*N* = 1132)	Unmarried/divorced	323	28.53
	Married	809	71.47
Education level (*N* = 1132)	Junior College (diploma)	192	16.96
	Bachelor's degree	912	80.57
	Master's degree	28	2.47
	PhD	6	0.53
Work duration (years) (*N* = 1132)	< 5	231	20.41
	5–10	375	33.13
	11–15	316	27.92
	16–19	92	8.13
	≥ 20	118	10.42
Title (*N* = 1132)	Nurse	207	18.29
	Nurse Practitioner	438	38.69
	Supervising Nurse Practitioner	457	40.37
	Associate Director of Nurse Practitioners	30	2.65
Ranking (*N* = 1132)	N0	91	8.04
	N1	218	19.26
	N2	435	38.43
	N3	388	34.28
Position (*N* = 1132)	Nurse	1039	91.78
	Nurse Manager	81	7.16
	Section Nurse Manager	12	1.06
Unit establishment (*N* = 1132)	Contractual	912	80.57
	Personnel agency	55	4.86
	Official establishment	165	14.58
Salary level (in months CNY) (*N* = 1132)	< 4000	136	12.01
	4000–7999	820	72.44
	≥ 8000	176	15.55
Section (*N* = 1132)	Internal medicine	361	31.89
	Surgery	286	25.27
	Outpatient	138	12.19
	Operating room	82	7.24
	Others	265	23.41

*Note:* *N*‐value represents the effective sample size of the project. *Source:* Authors.

### 3.2 Model Design and Variable Description

#### 3.2.1 Variable Description

##### 3.2.1.1 Dependent Variable (DV)

The dependent variables of this study are the EVLN behaviours. We utilised the four‐dimensional behaviour scale developed by Farrell (1983) and applied the CRITIC weighting method to measure these behaviours (Diakoulaki et al. 1995). Specifically, we used the CRITIC weighting method to calculate the weight of each indicator (see Table 2) and then formed the dependent variable value for each secondary indicator factor through weighted summation. The indicator values are presented in Table 2.

**TABLE 2:** EVLN behavioural indicators of nurses in hospital.

First–level indicators	Second–level indicators	Sign	Weight
Exit behaviour	I often feel like quitting my job	+	31.57%
	I often feel like quitting my current job	+	26.62%
	I would accept another new job	+	28.39%
	I would quit my current job	+	13.42%
Voice behaviour	I often discuss the current situation with my superiors	+	29.63%
	I often make suggestions to my superiors about certain problems in the organisation	+	19.48%
	I often suggest solutions to problems in the way leaders in my department supervise or direct work	+	25.82%
	Outside of work, I often ask my colleagues if they need help	+	25.07%
Loyalty behaviour	I often think that my work is the best	+	27.25%
	I will not leave the organisation regardless of whether it is in good times or bad times	+	28.71%
	I will always do my best to protect the reputation of the organisation when others criticise it	+	25.34%
	I will not leave until the problems in the organisation are resolved	+	18.70%
Neglect behaviour	Sometimes, when I don't feel like working, I will call in sick or find other reasons not to work	+	21.65%
	If I am not in the mood to work, I may be late for work	+	13.57%
	I will not work at full strength	+	23.28%
	I will often take breaks during work or not work at all	+	16.31%
	I may lose motivation for work	+	25.19%

*Note:* A five‐point Likert scale was employed for all items (1 = “strongly disagree”, 2 = “disagree”, 3 = “neutral”, 4 = “agree”, 5 = “strongly agree”). The questionnaires were anonymous, and respondents were asked to independently, conscientiously, and responsibly select the EVLN behaviours corresponding to the 17 items. *Source:* Authors.

##### 3.2.1.2 Independent Variable (IV)

The independent variable of this study is nurses' job dissatisfaction (*NJD*). We utilised the job dissatisfaction scale developed by Turnbach et al. (2024), and applied the combined assignment method to measure nurses' job dissatisfaction. Initially, the APH hierarchical method was employed to calculate the weights of the three‐level indicators. Subsequently, the entropy method was used to calculate the weights of the secondary indicators. Finally, the combined assignment method was used to compute the weighted sum of the values of nurses' job dissatisfaction. The values of the indicators are presented in Table 3.

**TABLE 3:** Job dissatisfaction indicators of nurses in hospital.

First–level indicators	Second–level indicators	Third–level indicators	Sign	Weight
Nurse job dissatisfaction	Internal job dissatisfaction	Emotional exhaustion and burnout	+	9.05%
		Feelings of ineffectiveness	+	6.17%
		Lack of autonomy	+	12.82%
		Compassion fatigue	+	12.14%
		Conflicting personal values	+	14.76%
		Other internal reasons	+	9.21%
	External job dissatisfaction	Insufficient professional development opportunities	+	4.36%
		Poor work‐life balance	+	7.28%
		Heavy workload and understaffing	+	5.87%
		Inadequate compensation	+	6.12%
		Lack of administrative support	+	7.83%
		Other external reasons	+	4.39%

*Note:* The scale utilises a five‐point Likert scale for measurement (1 = “strongly disagree”, 2 = “disagree”, 3 = “neutral”, 4 = “agree”, 5 = “strongly agree”). All questionnaires were completed anonymously, and respondents were required to independently, conscientiously, and responsibly select their level of satisfaction for each of the five items. *Source:* Authors.

##### 3.2.1.3 Control Variables (CV)

Based on the literature, several factors such as working years, professional title, ranking, and others impact nurses' behavioral responses, including Exit, Voice, Loyalty, and Neglect (EVLN) behaviors (Blegen 1993; Aiken et al. 2002; Tourangeau and Cranley 2006; Laschinger et al. 2009; McHugh and Lake 2010; Cowden and Cummings 2012). Drawing on existing research, we selected working years (WY), professional title (Title), ranking (Rank), position (Posit), unit establishment (UE), salary level (Salary), and section (Sec) as control variables. The specific definitions of each variable are provided in Table 4.

**TABLE 4:** Variable definition table.

Variable type	Variable name	Symbol	Measurement method
Dependent variable	Exit behaviour	Exit	According to the CRITIC weighting method of EVLN Scale, it is calculated comprehensively Exit = ⅀(three‐level indicators affecting Exit × Weight)
	Voice behaviour	Voice	According to the CRITIC weighting method of EVLN Scale, it is calculated comprehensively Voice = ⅀(three‐level indicators affecting Voice × Weight)
	Loyalty behaviour	Loyalty	According to the CRITIC weighting method of EVLN Scale, it is calculated comprehensively Loyalty = ⅀(three‐level indicators affecting Loyalty × Weight)
	Neglect behaviour	Neglect	According to the CRITIC weighting method of EVLN Scale, it is calculated comprehensively Neglect = ⅀(three‐level indicators affecting Neglect × Weight)
Independent variable	Nurse job dissatisfaction	NJD	The APH hierarchical analysis method was employed to comprehensively calculate the nurse job dissatisfaction scale NJD = Internal Nurses' Job Dissatisfaction (INJD) × Weight + External Nurses' Job Dissatisfaction (ENJD) × Weight
Control variables	Work duration (in years)	WY	< 5 = 1, 5–10 = 2, 11–15 = 3, 16–19 = 4, ≥ 20 = 5
	Title	Title	Nurse = 1, Nurse Practitioner = 2, Supervising Nurse Practitioner = 3, Associate Director of Nurse Practitioners = 4
	Ranking	Rank	N0 = 1, N1 = 2, N2 = 3, N3 = 4
	Position	Posit	Nurse = 1, Nurse Manager = 2, Section Nurse Manager = 3
	Unit Establishment	UE	Contractual = 1, Personnel Agency = 2, Official Establishment = 3
	Salary level	Salary	< 4000 = 14,000–7999 = 2, ≥ 8000 = 3
	Section	Sec	Virtual variables: Internal medicine = 1, Surgery = 2, others = 0

#### 3.2.2 Model Design

To test research Hypotheses 1a, 1b, 1c and 1d, we constructed the following regression model (3–1):

(3–1) $${\text{EVLN}}_{\mathrm{it}}=\beta_{1}+\beta_{2}\;{\mathrm{NJD}}_{\mathrm{it}}+\beta_{3}⅀{\text{Control}}_{\mathrm{it}}+\beta_{4}⅀\mathrm{Sec}+\alpha_{i}t$$

In model (3–1), EVLN is the dependent variable (DV), respectively representing the exit, voice, loyalty, and neglect behaviour of nurses in hospital; NJD is the independent variable (IV), representing the job dissatisfaction of nurses in hospital; Control is a series of control variables (CV); Sec is the fixed effect, representing the fixed effect of the section; *α* is the random disturbance term; subscripts *i* and *t* represent individual nurses and time, respectively.

To test research Hypotheses 2a, 2b, 2c and 2d, the study constructed the following regression model (3–2):

(3‐2) $${\text{VNEE}}_{\mathrm{it}}=\beta_{1}+\beta_{2}\;{\text{LVNV}}_{\mathrm{it}}+\beta_{3}⅀{\text{Control}}_{\mathrm{it}}+\beta_{4}⅀\mathrm{Sec}+\alpha_{i}t$$

In model (3–1), VNEE is the dependent variable (DV), respectively representing the voice, neglect, exit, and exit behaviour of nurses in hospital in light of the order of research hypotheses; LVNV is the independent variable (IV), respectively representing the loyalty, voice, neglect, and voice behaviour of nurses in hospital in view of the order of research hypotheses; Control is a series of control variables (CV); Sec is the fixed effect, representing the fixed effect of the section; *α* is the random disturbance term; subscripts *i* and *t* represent individual nurses and time, respectively.

#### 3.2.3 Statistical and Analytical Methods

This study employed SPSS V29.0, Stata 17.0, SmartPLS 4.0, and SPSSAU software to analyze the valid survey data and perform empirical analyses using quantitative methods.

## 4 Results

### 4.1 Descriptive Statistics and Reliability Checks

Descriptive statistics and reliability tests are provided in Table 5. Before conducting descriptive statistics, we assessed the validity of the measurement model by calculating the composite reliability (CR) and average variance extracted (AVE) for each construct. The results show that the CR values for all measurement constructs exceed 0.9 (Nunnally 1967; Babbie 1975), and the AVE values are greater than 0.5, indicating that the measurement model demonstrates good convergent validity, internal consistency, stability, and reliability. In the descriptive statistics, the mean and standard deviation of loyalty behaviour and voice behaviour are higher than those of neglect behaviour and exit behaviour. Additionally, the mean and standard deviation of internal nurses' job dissatisfaction are higher than those of external nurses' job dissatisfaction (Mean: 1.4143 > 0.9772; SD: 0.5936 > 0.4623). Nurses experiencing dissatisfaction tend to first stay loyal, then speak up, disengage, or exit. From this we can know that when nurses in tertiary public hospitals in Chongqing's urban and county districts experience job dissatisfaction, their initial response is typically one of observation, waiting for the organisation to address their legitimate concerns. During this phase, their behaviour reflects a form of loyalty. If no improvements are observed over time, they shift to expressing their concerns through voice behaviour. If their voice behaviour remains unheeded, they resort to neglect, passively reducing their work effort (note: or, alternatively, they may directly choose to exit the organisation). In the end, if neglect does not lead to satisfaction, they opt for exit behaviour in search of better career opportunities. This sequence of behaviours provides preliminary support for research Hypotheses 2a, 2b, 2c and 2d, and aligns with the findings of Lin et al. (2010).

**TABLE 5:** Descriptive statistics and reliability checks.

Variables	Obs	Mean	SD	Min	Median	Max	(Cronbach's Alpha)
Exit	1132	3.4226	0.2823	0.9999	3.2331	4.9995	0.974
Voice	1132	4.1465	1.2676	3.2638	4.0000	5.0000	0.955
Loyalty	1132	4.4898	1.3134	3.9920	4.4996	5.0000	0.959
Neglect	1132	3.7615	0.4331	1.0000	4.0000	5.0000	0.973
INJD	1132	1.4143	0.5936	0.5472	1.6416	2.7360	0.957
ENJD	1132	0.9772	0.4623	0.4528	0.9056	2.2640	0.923

In the descriptive statistics, the mean of loyalty behaviour (*Loyalty*) is 4.4898 with a median of 4.4996 and a standard deviation of 1.3134, showing significant variability in loyalty behaviour among the nurses in the sample. The mean of voice behaviour (*Voice*) is 4.1465, with a median of 4.0000 and a standard deviation of 1.2676, indicating remarkable variation in voice behaviour across the sampled nurses. Conversely, variables such as neglect behaviour (*Neglect*), exit behaviour (*Exit*), internal nurses' job dissatisfaction (*INJD*), and external nurses' job dissatisfaction (*ENJD*) exhibit small standard deviations, presenting little variation in these indicators within the sample.

### 4.2 Exploratory Factor Analysis, Correlation Analysis and Common Method Bias (CMB) Test

When conducting the correlation analysis, an exploratory factor analysis (EFA) (Hotelling 1933) was initially performed to determine whether each question was adequately and scientifically represented within each factor. The results indicated that most of the 29 questions aligned well with the theoretical dimensions, were reasonably and scientifically reflected in the six factor dimensions with a cumulative variance explanation rate of 91.298%, and with only a few cases exhibiting cross‐loadings or negative loadings.

Subsequently, a Pearson correlation analysis was performed to examine potential collinearity among the variables. The correlation analysis results in Table 6 indicate that nurses' job dissatisfaction (*NJD*) is significantly positively correlated with exit behaviour (*Exit*) and neglect behaviour (*Neglect*) at the 0.01 level, supporting the Hypotheses 1a and 1b. Additionally, nurses' job dissatisfaction (*NJD*) is significantly negatively related to voice behaviour (*Voice*) and loyalty behaviour (*Loyalty*) at the 0.01 level, supporting the Hypotheses 1c and 1d.

**TABLE 6:** Correlations between key study variables.

6–1 Correlations analysis (Exit behaviour)									
	Exit	NJD	WY	Title	Rank	Posit	UE	Slary	Sec
Exit	1								
NJD	0.1267***	1							
WY	0.0131	−0.0210	1						
Title	0.0538*	−0.0855***	0.6582***	1					
Rank	0.0304	−0.0655**	0.7090***	0.7406***	1				
Posit	0.0639**	−0.0442	0.3233***	0.3518***	0.2747***	1			
UE	−0.0423	0.0348	0.5077***	0.4263***	0.3218***	0.4119***	1		
Slary	0.0642**	−0.1155***	0.3802***	0.4355***	0.4364***	0.3459***	0.3061***	1	
Sec	−0.0274	−0.0016	−0.0320	−0.0257	0.0071	0.0057	0.0162	0.0874***	1

6–2 Correlations analysis (voice behaviour)									
	Voice	NJD	WY	Title	Rank	Posit	UE	Slary	Sec
Voice	1								
NJD	−0.1694***	1							
WY	0.0745**	−0.0210	1						
Title	0.0670**	−0.0855***	0.6582***	1					
Rank	0.1072***	−0.0655**	0.7090***	0.7406***	1				
Posit	0.0296	−0.0442	0.3233***	0.3518***	0.2747***	1			
UE	−0.0235	0.0348	0.5077***	0.4263***	0.3218***	0.4119***	1		
Slary	0.0354	−0.1155***	0.3802***	0.4355***	0.4364***	0.3459***	0.3061***	1	
Sec	−0.0030	−0.0016	−0.0320	−0.0257	0.0071	0.0057	0.0162	0.0874***	1

6–3 Correlations analysis (loyalty behaviour)									
	Loyalty	NJD	WY	Title	Rank	Posit	UE	Slary	Sec
Loyalty	1								
NJD	−0.2937***	1							
WY	0.0363	−0.0210	1						
Title	0.0519*	−0.0855***	0.6582***	1					
Rank	0.0682**	−0.0655**	0.7090***	0.7406***	1				
Posit	0.0305	−0.0442	0.3233***	0.3518***	0.2747***	1			
UE	−0.0309	0.0348	0.5077***	0.4263***	0.3218***	0.4119***	1		
Slary	0.0550*	−0.1155***	0.3802***	0.4355***	0.4364***	0.3459***	0.3061***	1	
Sec	−0.0179	−0.0016	−0.0320	−0.0257	0.0071	0.0057	0.0162	0.0874***	1

6–4 Correlations analysis (neglect behaviour)									
	Neglect	NJD	WY	Title	Rank	Posit	UE	Slary	Sec
Neglect	1								
NJD	0.1928***	1							
WY	−0.0557*	−0.0210	1						
Title	0.0051	−0.0855***	0.6582***	1					
Rank	−0.0052	−0.0655**	0.7090***	0.7406***	1				
Posit	−0.0125	−0.0442	0.3233***	0.3518***	0.2747***	1			
UE	−0.1012***	0.0348	0.5077***	0.4263***	0.3218***	0.4119***	1		
Slary	0.0115	−0.1155***	0.3802***	0.4355***	0.4364***	0.3459***	0.3061***	1	
Sec	−0.0438	−0.0016	−0.0320	−0.0257	0.0071	0.0057	0.0162	0.0874***	1

* *p* < 0.1.

** *p* < 0.05.

*** *p* < 0.01.

To further verify the presence of multicollinearity, a variance inflation factor (VIF) test was conducted on all variables. If the Pearson test indicates that the correlation coefficient between variables is greater than 0.8, multicollinearity may be present. Additionally, if the VIF value exceeds 10, multicollinearity is confirmed. The results of the VIF test for this study showed that all variable values ranged between 1.017 and 2.914, which is below the critical value of 10. This confirms that the constructed model does not have multicollinearity problems.

Finally, since this study relies on self‐reported data from a cross‐sectional survey, we employed Harman's single‐factor test to assess potential common method bias (CMB). The results indicated that the variance explained by a single factor was less than 50%, suggesting that no significant common method bias exists. Additionally, to enhance the credibility of the results, we applied the marker variable technique to minimize the impact of measurement methods on the findings.

### 4.3 Benchmark Regression

Table 7 presents the results of the baseline regression analysis. Column (1) displays the regression results between nurses' job dissatisfaction (*NJD*) and exit behaviour (*Exit*) after controlling for department effects and incorporating control variables. Columns (2) and (3) respectively show the regression results between *NJD* and voice behaviour (*Voice*) and between *NJD* and loyalty behaviour (*Loyalty*), also after controlling for department effects and adding control variables. Column (4) presents the regression results between *NJD* and neglect behaviour (*Neglect*), similarly accounting for department effects and control variables.

**TABLE 7:** Benchmark regression.

Variables	(1)	(2)	(3)	(4)
	Exit	Voice	Loyalty	Neglect
NJD	0.3924***	−0.1483***	−0.1714***	0.6603***
	(4.21)	(−3.51)	(−6.41)	(6.38)
WY	−0.0032	0.0352	0.0072	−0.0751
	(−0.06)	(1.57)	(0.63)	(−1.53)
Title	0.1545**	−0.0247	−0.0051	0.1582**
	(2.03)	(−0.51)	(−0.36)	(3.19)
Rank	−0.0717	0.0523*	0.0225	−0.0224
	(−0.86)	(1.97)	(0.86)	(−0.18)
Posit	0.3614**	0.0231	0.0147	0.1335
	(2.34)	(0.76)	(0.58)	(0.85)
UE	−0.2146***	−0.0461*	−0.0186	−0.2405***
	(−3.31)	(−1.91)	(−1.53)	(−3.81)
Slary	0.1821**	−0.0336	0.0218	0.2426
	(2.08)	(−0.87)	(0.76)	(1.39)
Constant	2.6641***	4.2638***	4.7174***	2.7572***
	(8.23)	(46.18)	(82.27)	(8.35)
Sec	YES	YES	YES	YES
N	1132	1132	1132	1132
R^2^	0.04	0.06	0.11	0.07
Adj. R^2^	0.03	0.05	0.10	0.06

* *p* < 0.1.

** *p* < 0.05.

*** *p* < 0.01.

The results indicate that dissatisfied nurses are more likely to disengage or leave. This confirms the proposed behavioural patterns. Besides, dissatisfaction is linked to reduced commitment and speaking out, which supports our theoretical model.

### 4.4 Robustness Checks

To further test the reliability of the results, we employed two methods for robustness checks (RC): subsample regressions method (Cifci and Oliver 2018; Cao et al. 2023; Li, Chen, et al. 2024, Li, Wong, et al. 2024, Li et al. 2025) and remeasurement method (Li and Tian 2023; Zhou et al. 2024).

#### 4.4.1 Subsample Regressions Method

Subsample regression is a common method for measuring the robustness of test results (Cifci and Oliver 2018; Cao et al. 2023). Given that the nurse manager is a key factor affecting nurses' job dissatisfaction (Lackey and Antrum 2024), we used subsample regression to test the robustness of our research conclusions, overcome endogeneity, and eliminate the impact of differences in sample hospital characteristics. We reduced the sample by excluding nurse managers and section nurse managers to obtain a subsample. Additionally, to prevent multicollinearity, we removed the control variable Position (*Posit*). The regression results in Table 8 indicate that nurses' job dissatisfaction (*NJD*) is significantly positively correlated with exit behaviour (*Exit*) and neglect behaviour (*Neglect*) at the 0.01 level, and significantly negatively related to voice behaviour (*Voice*) and loyalty behaviour (*Loyalty*) at the 0.01 level. These robustness check results are consistent with the conclusions of this study, demonstrating that our research findings are robust.

**TABLE 8:** Robust checks (subsample regressions method).

Variables	(1)	(2)	(3)	(4)
	Exit	Voice	Loyalty	Neglect
NJD	0.3768***	−0.1275***	−0.1523***	0.5404***
	(3.93)	(−2.99)	(−5.77)	(5.92)
WY	0.0198	0.0168	0.0023	−0.0700
	(0.41)	(0.97)	(0.20)	(−1.40)
Title	0.1450*	−0.0217	−0.0014	0.1658**
	(1.94)	(−0.89)	(−0.09)	(2.14)
Rank	−0.0847	0.0545**	0.0133	−0.0311
	(−1.23)	(2.40)	(0.87)	(−0.45)
UE	−0.1905***	−0.0274	−0.0137	−0.2061***
	(−2.86)	(−1.29)	(−0.98)	(−3.13)
Slary	0.1590*	−0.0335	0.0125	0.1414
	(1.76)	(−1.08)	(0.66)	(1.52)
Costant	3.0082***	4.2802***	4.7130***	2.8224***
	(9.18)	(44.06)	(78.17)	(8.55)
Sec	YES	YES	YES	YES
*N*	1043	1043	1043	1043
*R* ^2^	0.04	0.06	0.10	0.07
Adj. *R* ^2^	0.02	0.04	0.09	0.05

* *p* < 0.1.

** *p* < 0.05.

*** *p* < 0.01.

#### 4.4.2 Remeasurement Method

To further address the endogeneity problem, this study employs the remeasurement method (Li and Tian 2023; Zhou et al. 2024) to mitigate its impact on the conclusions. A new nurse job dissatisfaction scale is utilized, incorporating sub‐factors that influence nurse job dissatisfaction, such as relationships, support, freedom from fear, and breaks (Lackey and Antrum 2024). This approach allows for a reevaluation of the impact of nurses' job dissatisfaction (*NJD*) on EVLN behaviour. The results in Table 9 demonstrate that *NJD* is significantly positively correlated with exit behaviour (*Exit*) and neglect behaviour (*Neglect*), while being significantly negatively correlated with voice behaviour (*Voice*) and loyalty behaviour (*Loyalty*). These findings further underscore the robustness of our research results.

**TABLE 9:** Robust checks (remeasurement method).

Variables	(1)	(2)	(3)	(4)
	Exit	Voice	Loyalty	Neglect
NJD	0.3673***	−0.1362***	−0.1413***	0.5923***
	(4.47)	(−3.95)	(−6.75)	(7.60)
WY	−0.0039	0.0247	0.0065	−0.0725
	(−0.09)	(1.53)	(0.61)	(−1.53)
Title	0.1449**	−0.0138	−0.0033	0.1520**
	(2.02)	(−0.60)	(−0.21)	(2.05)
Rank	−0.0637	0.0418*	0.0121	−0.0132
	(−0.97)	(1.92)	(0.83)	(−0.20)
Posit	0.3635**	0.0278	0.0119	0.1436
	(2.42)	(0.59)	(0.41)	(0.90)
UE	−0.2022***	−0.0373*	−0.0208	−0.2336***
	(−3.28)	(−1.91)	(−1.65)	(−3.86)
Slary	0.1790**	−0.0259	0.0102	0.1514*
	(2.10)	(−0.89)	(0.57)	(1.73)
Constant	2.5888***	4.2818***	4.7176***	2.5647***
	(7.89)	(45.64)	(80.62)	(7.72)
Sec	YES	YES	YES	YES
*N*	1132	1132	1132	1132
*R* ^2^	0.05	0.07	0.11	0.09
Adj. *R* ^2^	0.03	0.05	0.10	0.08

* *p* < 0.1.

** *p* < 0.05.

*** *p* < 0.01.

## 5 Heterogeneity Checks and EVLN Behaviours Evolution Study

### 5.1 Heterogeneity Checks

Numerous scholars have demonstrated that employees' job dissatisfaction, influenced by personality traits, exhibits variations across socio‐demographic variables such as gender, age, marital status, and education level (Metle 2001; Halkos and Bousinakis 2017; Bagheri Hosseinabadi et al. 2019). Moreover, the reasons for nurses' job dissatisfaction vary (Pressley and Garside 2023; Lackey and Antrum 2024). Therefore, this study considers gender, age, marital status, and education level as heterogeneity indicators to examine the disparities in nurses' job dissatisfaction (*NJD*) and EVLN behaviours.

#### 5.1.1 Gender's Heterogeneity

The study explores how nurses' job dissatisfaction (*NJD*) varies by gender due to different personality traits. To investigate this, nurses were divided into male (assigned 1) and female (assigned 0) groups. Table 10 illustrates that compared to the male, *NJD* in female is associated with increased exit and neglect behaviours (*Exit* and *Neglect*), while it decreases voice and loyalty behaviours (*Voice* and *Loyalty*).

**TABLE 10:** Gender heterogeneity.

Variables	(1)	(2)	(3)	(4)	(5)	(6)	(7)	(8)
	Exit	Exit	Voice	Voice	Loyalty	Loyalty	Neglect	Neglect
	Gender = 1	Gender = 0	Gender = 1	Gender = 0	Gender = 1	Gender = 0	Gender = 1	Gender = 0
NJD	0.1838	0.3763***	0.2166	−0.1600***	−0.0508	−0.1679***	0.7110	0.5328***
	(0.40)	(3.96)	(1.62)	(−3.70)	(−0.52)	(−6.33)	(1.67)	(5.83)
WY	−0.1301	−0.0005	0.0483	0.0230	−0.0891	0.0083	−0.0675	−0.0654
	(−0.33)	(−0.01)	(0.42)	(1.39)	(−0.97)	(0.77)	(−0.20)	(−1.33)
Title	0.3420	0.1192	−0.0319	−0.0086	−0.0350	0.0008	0.3004	0.1249
	(0.67)	(1.62)	(−0.20)	(−0.36)	(−0.35)	(0.05)	(0.59)	(1.63)
Rank	−0.0523	−0.0664	0.1170	0.0423*	0.1535*	0.0081	0.0344	−0.0228
	(−0.14)	(−0.97)	(0.96)	(1.89)	(1.81)	(0.53)	(0.09)	(−0.33)
Posit	0.4740	0.3500**	0.2934*	0.0192	0.2867	0.0058	−0.2300	0.1274
	(0.63)	(2.22)	(1.71)	(0.40)	(1.42)	(0.20)	(−0.33)	(0.76)
UE	−0.2230	−0.2065***	−0.2256**	−0.0328	−0.1751*	−0.0173	−0.1479	−0.2352***
	(−0.45)	(−3.26)	(−2.29)	(−1.62)	(−1.69)	(−1.34)	(−0.38)	(−3.71)
Slary	0.1151	0.2071**	−0.2000	−0.0202	−0.0466	0.0142	0.4349	0.1328
	(0.23)	(2.33)	(−1.06)	(−0.68)	(−0.36)	(0.78)	(1.01)	(1.44)
Constant	2.7195*	2.6628***	4.1505***	4.2386***	4.6745***	4.6896***	1.9299	2.8497***
	(1.95)	(7.78)	(10.94)	(43.98)	(22.77)	(76.59)	(1.60)	(8.15)
Sec	YES	YES	YES	YES	YES	YES	YES	YES
*N*	56	1076	56	1076	56	1076	56	1076
*R* ^2^	0.26	0.04	0.32	0.07	0.29	0.12	0.37	0.07
Adj. *R* ^2^	−0.04	0.03	0.03	0.05	0.00	0.10	0.11	0.05

* *p* < 0.1.

** *p* < 0.05.

*** *p* < 0.01.

#### 5.1.2 Age's Heterogeneity

The study investigates how nurses' age impacts their job dissatisfaction due to varying personality traits. Nurses were categorised into two age groups: under 30 years old (assigned 1) and over 30 years old (including 30 years old, assigned 0). Table 11 reveals that compared to the younger (under 30 years old), nurses' job dissatisfaction (*NJD*) in the older (over 30 years old) is linked to increased exit and neglect behaviours (*Exit* and *Neglect*), while it decreases voice behaviour (*Voice*).

**TABLE 11:** Age heterogeneity.

Variables	(1)	(2)	(3)	(4)	(5)	(6)	(7)	(8)	(9)
	Exit	Exit	Voice	Voice	Loyalty	Loyalty	Loyalty	Neglect	Neglect
	Age = 1	Age = 0	Age = 1	Age = 0	Age = 1	Age = 0	Total sample	Age = 1	Age = 0
NJD	0.1582	0.5398***	−0.0810	−0.1730***	−0.1257***	−0.1848***	−0.1720***	0.2388	0.7355***
	(1.02)	(4.71)	(−1.21)	(−3.38)	(−2.99)	(−5.76)	(−6.43)	(1.61)	(6.79)
NJD × Age							0.0295		
							(1.23)		
WY	0.2203*	−0.0090	0.0537	0.0289	0.0321	0.0047	0.0129	0.2045*	−0.0659
	(1.81)	(−0.16)	(1.06)	(1.47)	(0.95)	(0.37)	(1.13)	(1.67)	(−1.10)
Title	0.1964*	0.1381	0.0079	−0.0262	−0.0012	−0.0081	−0.0024	0.0472	0.1929**
	(1.67)	(1.48)	(0.18)	(−0.93)	(−0.04)	(−0.43)	(−0.15)	(0.39)	(1.99)
Rank	−0.1173	−0.0384	0.0458	0.0432	−0.0079	0.0370*	0.0164	0.0014	−0.0353
	(−1.19)	(−0.40)	(1.29)	(1.41)	(−0.34)	(1.78)	(1.09)	(0.01)	(−0.36)
Posit	−0.0731	0.4218***	−0.2638***	0.0382	0.0425	0.0052	0.0128	−0.5878	0.2199
	(−0.29)	(2.60)	(−3.69)	(0.78)	(0.29)	(0.18)	(0.46)	(−1.21)	(1.28)
UE	−0.1565	−0.1990***	0.0321	−0.0424*	0.0068	−0.0256*	−0.0207	0.0248	−0.2696***
	(−0.92)	(−2.89)	(0.68)	(−1.93)	(0.20)	(−1.81)	(−1.64)	(0.17)	(−3.89)
Slary	0.2225	0.1464	−0.0709	0.0097	−0.0045	0.0276	0.0125	0.2474*	0.0709
	(1.60)	(1.31)	(−1.38)	(0.27)	(−0.14)	(1.27)	(0.70)	(1.73)	(0.62)
Constant	2.9536***	2.3745***	4.4299***	4.2091***	4.6474***	4.6443***	4.6727***	3.1919***	2.5070***
	(5.64)	(5.21)	(30.54)	(31.81)	(27.68)	(52.75)	(73.43)	(4.75)	(5.51)
Sec	YES	YES	YES	YES	YES	YES	YES	YES	YES
*N*	433	699	433	699	433	699	1132	433	699
*R* ^2^	0.05	0.07	0.07	0.09	0.08	0.15	0.11	0.08	0.10
Adj. *R* ^2^	0.01	0.04	0.03	0.06	0.04	0.12	0.10	0.04	0.08

* *p* < 0.1.

** *p* < 0.05.

*** *p* < 0.01.

The regression results from columns (5) and (6) of Table 11 reveal significantly negative coefficients of job dissatisfaction (*NJD*) for nurses both over and under 30 years old at the 1% significance level. This suggests that job dissatisfaction (*NJD*) among nurses of different age groups can reduce the likelihood of loyalty behaviour (*Loyalty*). Subsequent inclusion of the interaction term (*NJD**×**Age*) did not yield a significant coefficient, indicating no substantial difference in the impact of job dissatisfaction (*NJD*) on loyalty behaviour (*Loyalty*) between nurses of different age groups.

#### 5.1.3 Marital Heterogeneity

Nurses' job dissatisfaction (*NJD*) can be influenced by objective environmental factors, such as family circumstances, which vary based on marital status. To assess the impact of marital heterogeneity on the research conclusions, this study categorised *NJD* in hospitals into two groups: the married group, assigned a value of 1, and the unmarried/divorced group, assigned a value of 0.

The research results in columns (1) through (4) of Table 12 indicate that, compared to unmarried or divorced nurses, job dissatisfaction (*NJD*) among married nurses can increase the occurrence of exit behaviour (*Exit*) and decrease the occurrence of voice behaviour (*Voice*).

**TABLE 12:** Marriage heterogeneity.

Variables	(1)	(2)	(3)	(4)	(5)	(6)	(7)	(8)	(9)	(10)
	Exit	Exit	Voice	Voice	Loyalty	Loyalty	Loyalty	Neglect	Neglect	Neglect
	Marrige = 1	Marrige = 0	Marrige = 1	Marrige = 0	Marrige = 1	Marrige = 0	Total Sample	Marrige = 1	Marrige = 0	Total Sample
NJD	0.4153***	0.2905	−0.1459***	−0.1061	−0.1657***	−0.1442***	−0.1389***	0.5800***	0.4129**	0.5120***
	(3.95)	(1.55)	(−3.08)	(−1.25)	(−5.64)	(−2.75)	(−4.61)	(5.68)	(2.38)	(5.10)
NJD × Marrige							−0.0289			0.0529
							(−1.38)			(0.85)
WY	−0.0171	−0.0654	0.0217	0.0253	0.0109	0.0143	0.0102	−0.0408	−0.2329*	−0.0764
	(−0.33)	(−0.58)	(1.15)	(0.66)	(0.89)	(0.55)	(0.94)	(−0.75)	(−1.97)	(−1.54)
Title	0.1031	0.2510*	−0.0115	−0.0230	−0.0048	−0.0157	−0.0016	0.0978	0.2881**	0.1445*
	(1.21)	(1.73)	(−0.44)	(−0.45)	(−0.27)	(−0.44)	(−0.10)	(1.08)	(2.01)	(1.93)
Rank	−0.0283	−0.1375	0.0355	0.0758	0.0091	0.0421	0.0146	0.0082	0.0104	−0.0168
	(−0.36)	(−1.06)	(1.38)	(1.64)	(0.51)	(1.46)	(0.98)	(0.10)	(0.08)	(−0.25)
Posit	0.3693**	0.1909	0.0307	−0.1397	0.0318	−0.1579*	0.0153	0.1852	−0.5464**	0.1232
	(2.22)	(0.61)	(0.61)	(−0.93)	(1.08)	(−1.69)	(0.54)	(1.06)	(−2.19)	(0.77)
UE	−0.1806**	−0.2457*	−0.0438**	−0.0109	−0.0371**	0.0476*	−0.0221*	−0.2594***	−0.1328	−0.2268***
	(−2.54)	(−1.90)	(−1.96)	(−0.22)	(−2.54)	(1.71)	(−1.74)	(−3.63)	(−0.99)	(−3.63)
Slary	0.1549	0.2317	0.0067	−0.0946	0.0289	−0.0366	0.0109	0.1146	0.1506	0.1336
	(1.47)	(1.47)	(0.20)	(−1.52)	(1.48)	(−0.96)	(0.62)	(1.06)	(0.91)	(1.50)
Constant	2.8389***	2.4629***	4.2613***	4.3507***	4.6975***	4.7733***	4.6884***	2.9941***	2.7126***	2.7845***
	(7.44)	(4.17)	(35.23)	(23.34)	(62.08)	(41.30)	(77.10)	(7.73)	(4.91)	(8.32)
Sec	YES	YES	YES	YES	YES	YES	YES	YES	YES	YES
*N*	814	318	814	318	814	318	1132	814	318	1132
*R* ^2^	0.06	0.06	0.08	0.07	0.14	0.12	0.11	0.07	0.11	0.07
Adj. *R* ^2^	0.03	0.00	0.05	0.02	0.12	0.07	0.10	0.05	0.06	0.05

* *p* < 0.1.

** *p* < 0.05.

*** *p* < 0.01.

From the regression results in columns (5) and (6) of Table 12, the coefficients for job dissatisfaction (*NJD*) among married nurses and unmarried/divorced nurses are significantly negative at the 1% level, suggesting that job dissatisfaction (*NJD*) in nurses with different marital statuses can reduce the occurrence of loyalty behaviour (*Loyalty*). Additionally, after including the interaction term (*NJD × Marriage*) in the regression, the coefficient for the interaction term was not significant, indicating no significant difference in the impact of job dissatisfaction (*NJD*) on reducing loyalty behaviour (*Loyalty*) between nurses of different marital statuses.

The regression results in columns (8) and (9) of Table 12 indicate that job dissatisfaction (*NJD*) among nurses with different marital statuses can promote neglect behaviour (*Neglect*). After adding the interaction term (*NJD × Marriage*) and performing the regression, the coefficient for the interaction term was not significant, suggesting that job dissatisfaction (*NJD*) among nurses with different marital statuses does not significantly differ in promoting neglect behaviour (*Neglect*).

#### 5.1.4 Heterogeneity of Education Level

Different education levels result in varying personality traits among nurses, which subsequently influence their job dissatisfaction. To examine the impact of educational heterogeneity on the study's conclusions, nurses with a bachelor's degree or higher were grouped into one category (Degree = 1), while those with less than a bachelor's degree were grouped into another category (Degree = 0).

The regression results in columns (1) and (2) of Table 13 indicate that the coefficient of nurses' job dissatisfaction (*NJD*) is significantly positive at the 1% level for the bachelor's degree or higher, and significantly positive at the 10% level for nurses with less than a bachelor's degree. This suggests that job dissatisfaction (*NJD*) among nurses with different educational backgrounds promotes exit behaviour (*Exit*). Moreover, the impact of nurses' job dissatisfaction (*NJD*) on promoting exit behaviour (*Exit*) is stronger for the bachelor's degree or higher compared to those with less education.

**TABLE 13:** Degree heterogeneity.

Variables	(1)	(2)	(3)	(4)	(5)	(6)	(7)	(8)	(9)	(10)
	Exit	Exit	Voice	Voice	Loyalty	Loyalty	Loyalty	Neglect	Neglect	Neglect
	Degree = 1	Degree = 0	Degree = 1	Degree = 0	Degree = 1	Degree = 0	Total Sample	Degree = 1	Degree = 0	Total Sample
NJD	0.3737***	0.4463*	−0.1592***	−0.0614	−0.1665***	−0.1254**	−0.1473***	0.5290***	0.6258***	0.4443***
	(3.66)	(1.94)	(−3.50)	(−0.65)	(−5.97)	(−2.04)	(−4.63)	(5.44)	(2.82)	(4.23)
NJD × Degree							−0.0164			0.1336**
							(−0.73)			(2.02)
WY	−0.0304	0.1727	0.0302	−0.0314	0.0058	0.0035	0.0051	−0.0848	0.0944	−0.0612
	(−0.57)	(1.65)	(1.64)	(−0.78)	(0.47)	(0.13)	(0.48)	(−1.51)	(0.90)	(−1.27)
Title	0.0966	0.1439	−0.0205	0.0896	−0.0066	0.0159	−0.0008	0.0862	0.1847	0.1210
	(1.16)	(0.78)	(−0.78)	(1.63)	(−0.38)	(0.33)	(−0.05)	(1.00)	(0.99)	(1.57)
Rank	−0.0668	−0.1145	0.0451*	0.0138	0.0141	0.0038	0.0133	−0.0328	−0.0629	−0.0245
	(−0.91)	(−0.71)	(1.86)	(0.25)	(0.87)	(0.09)	(0.89)	(−0.43)	(−0.38)	(−0.36)
Posit	0.3392**	0.6680	0.0340	−0.0360	0.0111	−0.0109	0.0144	0.0982	0.6752	0.1323
	(2.07)	(1.25)	(0.67)	(−0.24)	(0.36)	(−0.13)	(0.51)	(0.57)	(1.31)	(0.82)
UE	−0.1533**	−0.4561***	−0.0324	−0.0673	−0.0162	−0.0269	−0.0200	−0.1722**	−0.5886***	−0.2268***
	(−2.23)	(−2.98)	(−1.49)	(−1.39)	(−1.15)	(−0.83)	(−1.56)	(−2.57)	(−3.57)	(−3.68)
Slary	0.1842*	0.0663	−0.0331	0.0008	0.0132	−0.0030	0.0120	0.1433	0.0234	0.1308
	(1.91)	(0.33)	(−1.02)	(0.01)	(0.68)	(−0.07)	(0.68)	(1.43)	(0.11)	(1.47)
Constant	2.9146***	1.9131**	4.2973***	4.1751***	4.7157***	4.7116***	4.7004***	3.0817***	1.8168**	2.8124***
	(8.24)	(2.15)	(40.12)	(17.22)	(70.43)	(34.00)	(78.72)	(8.47)	(2.07)	(8.41)
Sec	YES	YES	YES	YES	YES	YES	YES	YES	YES	YES
*N*	910	222	910	222	910	222	1132	910	222	1132
*R* ^2^	0.04	0.10	0.08	0.12	0.13	0.11	0.11	0.06	0.16	0.07
Adj. *R* ^2^	0.02	0.03	0.06	0.05	0.11	0.04	0.10	0.04	0.09	0.06

* *p* < 0.1.

** *p* < 0.05.

*** *p* < 0.01.

The regression results in columns (3) and (4) of Table 13 indicate that nurses with an undergraduate degree or higher experience greater reductions in voice behaviour (*Voice*) due to job dissatisfaction (*NJD*) compared to those with less education. Additionally, columns (5) and (6) show that the coefficient for job dissatisfaction (NJD) is significantly negative at the 1% level for nurses with an undergraduate degree or higher, and at the 5% level for those with less education. This suggests that nurses' job dissatisfaction (*NJD*) leads to a reduction in loyalty behaviour (*Loyalty*) across different educational backgrounds, with a more pronounced effect for nurses with an undergraduate degree or higher.

The regression results in columns (8) and (9) of Table 13 show that the coefficients for job dissatisfaction (*NJD*) are significantly positive at the 1% level for both nurses with a bachelor's degree or higher and those with less than a bachelor's degree. This indicates that job dissatisfaction (*NJD*) across different educational backgrounds can increase the occurrence of neglect behaviour (*Neglect*). Furthermore, when the interaction term (*NJD×Degree*) is included in the model, its regression coefficient is significantly positive, suggesting that job dissatisfaction (*NJD*) is more likely to promote neglect behaviour (*Neglect*) among nurses with a bachelor's degree or higher compared to those with less education.

### 5.2 Study on the Evolution of EVLN Behaviours in Relation to Nurses' Job Dissatisfaction

In terms of the internal evolution of hospital nurses' EVLN behaviours, the data in Table 14 show that loyalty behaviour has a positive impact on voice behaviour, voice behaviour positively influences neglect behaviour, neglect behaviour positively impacts exit behaviour, and voice behaviour also has a positive impact on exit behaviour. Therefore, Hypotheses 2a, 2b, 2c and 2d are all confirmed. This indicates that the internal evolution of hospital nurses' EVLN behaviours, from loyalty to exit, generally follows the path of “loyalty behavior → voice behavior → neglect behavior → exit behavior.”

**TABLE 14:** Hypothesis testing results of EVLN behaviours.

Hypotheses	Relationships between variables	Standardised path coefficients (*β*)	Standard error (STDEV)	*T*‐value	*p*	Results
Hypothesis 2a	Loyalty→Voice	0.611	0.023	26.965	0.000 (***)	Accepted
Hypothesis 2b	Voice→Neglect	0.005	0.031	3.171	0.024 (**)	Accepted
Hypothesis 2c	Neglect→Exit	0.770	0.020	39.366	0.000 (***)	Accepted
Hypothesis 2d	Voice→Exit	0.065	0.020	3.216	0.001 (***)	Accepted

** *p* < 0.05.

*** *p* < 0.01.

Specifically, the transition of hospital nurses from loyalty to exit behaviour can be divided into two main stages:

The first step is the transition from loyalty behaviour to voice behaviour. The findings of this study align with the conclusions of Hirschman (1970) and Farrell (1983), indicating a positive correlation between loyalty and voice behaviour. A key distinction in this study is that loyalty behaviour is not directly linked to exit behaviour. When employees experience job dissatisfaction, they initially remain silent, hoping for improvement. If the situation does not improve, they will opt for voice behaviour rather than exit.

The second step involves the transition from voice behaviour to exit behaviour. Specifically, the results of the model (3–2) analysis show that when voice behaviour is not addressed, hospital nurses may choose to exit directly. Furthermore, the results indicate that voice behaviour is positively correlated with neglect behaviour, which in turn is positively correlated with exit behaviour, suggesting that neglect behaviour serves as a mediating variable between voice and exit behaviours. When nurses in hospital express dissatisfaction with their work and the organisation fails to address these concerns, they may develop negative attitudes toward their job, exhibit a careless work attitude, and ultimately seek opportunities to leave. If voice behaviour fails, neglect behaviour often follows, and this neglect may ultimately lead to exit. This demonstrates that if voice behaviour is not addressed, nurses are likely to engage in exit behaviour.

**FIGURE 3:**
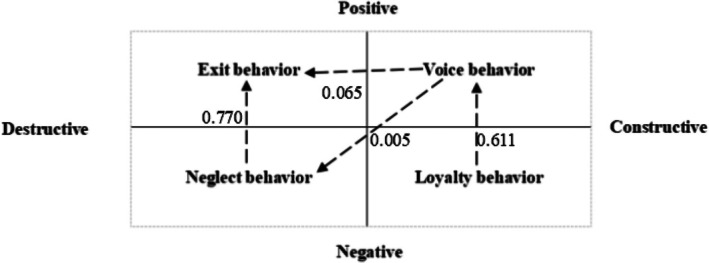
Internal evolution path of Exit, Voice, Loyalty, and Neglect (EVLN) behaviours.

In summary, after choosing loyalty behaviour, if nurses in hospital see no improvement in their dissatisfaction, they will resort to voice behaviour. If their voice behaviour is still not addressed, they will either choose to exit directly or transition to exit behaviour through neglect (see Figure 3).

## 6 Conclusion

### 6.1 Research Findings

Based on the principles of dynamic game theory, this study developed a theoretical model to examine the relationship between nurse job dissatisfaction (NJD) and EVLN behavioural responses in Chongqing's public tertiary hospitals, as well as the evolution of these behaviours.

The empirical findings reveal the following: First of all, nurse job dissatisfaction (NJD) is positively correlated with exit behaviour and neglect behaviour, while it is negatively correlated with voice behaviour and loyalty behaviour. These results align with the predictions of dynamic game theory (Haurie et al. 2012; Başar and Zaccour 2018; Ke et al. 2022), indicating that the relationship between job dissatisfaction and EVLN behaviours among nurses in Chongqing's public hospitals is consistent with findings from studies on corporate governance. This suggests that a certain level of commonality exists between corporate governance and hospital management, allowing both fields to benefit from the insights of one another's research.

And second, the heterogeneity analysis shows that the impact of nurse job dissatisfaction on EVLN behaviours varies across gender, age, marital status, and education level. Specifically, complete heterogeneity is observed in gender and education level, while partial heterogeneity is found in age and marital status. These findings are largely consistent with previous research, indicating that job dissatisfaction, influenced by personality traits, is heterogeneous across socio‐demographic variables such as gender, age, marital status, and education level (Metle 2001; Bagheri Hosseinabadi et al. 2019; Halkos and Bousinakis 2017), which further leads to differences in the reasons for nurse job dissatisfaction (Pressley and Garside 2023; Lackey and Antrum 2024).

Thirdly, according to the evolutionary analysis of EVLN behaviours, nurses in hospitals tend to choose voice behaviour following loyalty behaviour, followed by neglect and exit behaviours. This pattern aligns with the Confucian cultural emphasis on “moderation and stability,” which often leads hospital nurses to be reluctant to leave institutions where they have worked for many years. Under the influence of historical inertia and traditional values, nurses in hospitals typically wait for a period when dissatisfied with their work. If the situation does not improve, they actively engage in communication with the hospital to remain employed. This phenomenon is more evident in highly competitive market environments and during economic downturns. Only when communication fails or nurses are compelled by circumstances do they opt for exit behaviour.

### 6.2 Managerial Implication

The findings of this study have significant practical and managerial implications. The results indicate that nurses' job dissatisfaction is a key factor driving behaviours such as exit, voice, loyalty, and neglect. Therefore, healthcare policymakers, hospital administrators, and nursing practitioners should implement targeted interventions to address nurses' job dissatisfaction. Given the different manifestations of nurses' EVLN behaviours, hospitals should develop proactive intervention strategies to minimise the occurrence of exit and neglect behaviours while promoting voice and loyalty behaviours. Nursing practitioners should recognise the root causes of their job dissatisfaction and regularly assess and address negative emotions. Hospital administrators should establish effective communication and feedback mechanisms, break down hierarchical reporting barriers, and foster a more open and supportive organisational culture, aiming to create a people‐oriented management model. Specifically, measures such as psychological counselling, performance reforms, skills training, and career advancement opportunities can be implemented to improve the work environment and reduce nurses' job dissatisfaction. Additionally, healthcare policymakers should investigate the underlying causes of nurses' job dissatisfaction and their EVLN responses. Effective measures should be taken at the institutional and policy distribution levels to design relevant health policies that promote the scientific allocation and sustainable development of regional healthcare resources.

### 6.3 Theoretical Implications

This study makes several contributions to the existing literature. First, by incorporating the Chinese cultural context, this research introduces dynamic game theory into the analysis framework of EVLN behaviour to explore the relationship between nurses' job dissatisfaction and EVLN responses. This framework not only enriches the application of EVLN theory within China and the broader Chinese cultural sphere, but also provides new empirical support for hospital management regarding how nurses' job dissatisfaction leads to various EVLN responses. Unlike most studies conducted in Western countries, this research examines the influence of hierarchical structures in Chinese culture on nurses' EVLN behaviour choices when faced with job dissatisfaction, particularly the selection of voice behaviour. This highlights that, under the influence of Chinese collectivist culture, nurses are more likely to cope with job dissatisfaction through loyalty rather than directly expressing dissatisfaction, thus demonstrating the significant impact of cultural differences on EVLN behaviours. Second, this study offers a new application of Farrell's EVLN theory (Pendola 2024), specifically addressing the responses and degrees of EVLN behaviours in relation to nurses' job dissatisfaction. It provides a medical version of the theoretical model, expanding the scope of this theory and further refining it by analysing the heterogeneity of variables such as gender, age, marital status, and education level. Additionally, this study integrates perspectives from psychology, behavioural economics, and hospital management, offering new insights for the advancement of these fields. Finally, this study is the first to apply dynamic game theory to investigate the evolving mechanisms of EVLN behaviours triggered by nurses' job dissatisfaction, thus filling a gap in the literature and providing new theoretical support for the development of behaviour mechanism models in hospital management.

### 6.4 Limitations and Future Directions

We must acknowledge the limitations of this study and propose directions for future research. First, our study utilized cross‐sectional data, which allows us to capture only the current responses of nurses' EVLN behaviours to job dissatisfaction, without revealing long‐term trends. Second, this study relied solely on self‐reported data, which may be subject to social desirability bias. Additionally, the Nurses' Job Dissatisfaction Scale considered only a subset of key factors when designed, whereas the factors contributing to nurses' job dissatisfaction are numerous (Pressley and Garside 2023; Lackey and Antrum 2024). Therefore, future research should incorporate additional factors contributing to job dissatisfaction to enhance the reliability of the findings. Third, this study is limited to tertiary public hospitals in Chongqing and does not include data from other provinces or cities in China. Consequently, the generalizability of the findings remains uncertain. To improve external validity, future research should collect data from public hospitals in other regions for comparison. Fourth, this study focused only on the internal pathways of EVLN behaviours triggered by nurses' job dissatisfaction, without considering how structural differences in job dissatisfaction might affect the evolution of these behaviours. Future research should account for these factors to control the occurrence of nurses' dissatisfaction behaviours, thereby improving the comprehensiveness and accuracy of the research. Finally, due to the unique nature of China's healthcare political system (Blumenthal and Hsiao 2005; Jakovljevic et al. 2023) and limitations in the research conditions, this study did not include data from public hospitals in other countries. Therefore, whether the conclusions of this study can be generalized to countries outside of China requires further validation. Future research should collaborate with scholars from other countries to explore the work environments of nurses globally, contributing to the sustainable development of healthcare management theory worldwide.

## Author Contributions

Jackie Zhanbiao Li analysed data and drafted the manuscript. Wanqin Hu designed the study and performed the data collection. Yingqian Lao and Ming Chen made substantive intellectual contributions to the conception of the work and the interpretation of the data and revised the manuscript. These authors contributed equally to this work.

## Ethics Statement

Approval was obtained from the ethics committee of Ophthalmic & Dental Center of Putuo District in Shanghai (No. 2023‐03). All research was performed in accordance with the relevant guidelines and regulations, including the Declaration of Helsinki.

## Consent

Informed consent was obtained from all individual participants included in the study.

## Conflicts of Interest

The authors declare no conflicts of interest.

## Data Availability

Data will be made available on request.
